# Nondestructive natural gas hydrate recovery driven by air and carbon dioxide

**DOI:** 10.1038/srep06616

**Published:** 2014-10-14

**Authors:** Hyery Kang, Dong-Yeun Koh, Huen Lee

**Affiliations:** 1 Department of Chemical and Biomolecular Engineering, Korea Advanced Institute of Science and Technology, 291 Daehak-ro, Guseong-dong, Yuseong-gu, Daejeon 305-701, South Korea; 2 Graduate School of EEWS, Korea Advanced Institute of Science and Technology, 291 Daehak-ro, Guseong-dong, Yuseong-gu, Daejeon 305-701, South Korea

## Abstract

Current technologies for production of natural gas hydrates (NGH), which include thermal stimulation, depressurization and inhibitor injection, have raised concerns over unintended consequences. The possibility of catastrophic slope failure and marine ecosystem damage remain serious challenges to safe NGH production. As a potential approach, this paper presents air-driven NGH recovery from permeable marine sediments induced by simultaneous mechanisms for methane liberation (NGH decomposition) and CH_4_-air or CH_4_-CO_2_/air replacement. Air is diffused into and penetrates NGH and, on its surface, forms a boundary between the gas and solid phases. Then spontaneous melting proceeds until the chemical potentials become equal in both phases as NGH depletion continues and self-regulated CH_4_-air replacement occurs over an arbitrary point. We observed the existence of critical methane concentration forming the boundary between decomposition and replacement mechanisms in the NGH reservoirs. Furthermore, when CO_2_ was added, we observed a very strong, stable, self-regulating process of exchange (CH_4_ replaced by CO_2_/air; hereafter CH_4_-CO_2_/air) occurring in the NGH. The proposed process will work well for most global gas hydrate reservoirs, regardless of the injection conditions or geothermal gradient.

Natural gas hydrates (NGHs) are solid, non-stoichiometric compounds containing a large amount of natural gas in deep sea sediments or permafrost regions and they have strong potential as a next-generation energy source^1^,^2^. How to efficiently and safely exploit the tremendous amount of natural gas stored as gas hydrate underneath the surface of the earth remains an urgent challenge, and fortunately several field trials for NGH production have suggested a hopeful new prospect for using gas hydrates as energy resources. Recently, one onshore field trial was carried out in 2012 on the Alaska North Slope in the United States of America^3^, and the first offshore field trial was attempted in 2013 in the Eastern Nankai Trough in Japan^4^,^5^. Meanwhile, in Korea, two Ulleung Basin Gas Hydrate (UBGH) Drilling Expeditions were carried out (2007 and 2010), and they confirmed the existence of considerable amounts of NGH in the deep-sea sediments there^6^,^7^,^8^. A UBGH field production test is scheduled for mid-2015 at the sites (UBGH 2–6) with geological conditions providing the greatest potential^8^.

The currently proposed technologies for marine NGH production are generally derived from standard methods used in conventional oil and gas industries. These take advantage of the driving potential of differences in temperature, pressure and chemical potential, which cause significant changes in the conditions within NGH reservoirs. Thermal stimulation causes vacillation between NGH formation and dissociation temperatures (ΔT), depressurization shifts the reservoir pressure to much lower than NGH-formation pressure (ΔP), and inhibitor injection alters the chemical environment (Δμ) of the NGH to inhibit the stable formation of gas hydrates^9^. However, these methods are all dissociation-based technologies, which can raise serious concerns about repercussions such as slope failure and seabed ecosystem destruction^10^. A CH_4_-replacement mechanism driven by CO_2_ injection has recently been suggested as a plausible new approach to production^11^,^12^. It has the potential of being a nondestructive means by which energy production and carbon dioxide sequestration might be achieved simultaneously. However, under the harsh conditions of naturally occurring gas hydrate sites, injected CO_2_ gas readily transforms to a liquid state, slowing and weakening its soaking and migration. Undesirable fluctuation between the two CO_2_ phases (gas and liquid) makes injection and diffusion very unstable, which can possibly lead to a low replacement rate and recovery yield. To overcome such weaknesses, in previous reports, we proposed the use of a gaseous mixture of N_2_ and CO_2_^13^,^14^,^15^. The overall recovery of methane was greatly improved by specific attack of CH_4_ molecules in the small cages of structure (sI) with added N_2_ molecules. In a field production test on the Alaska North Slope in 2012, the CO_2_/N_2_ injection method was adopted as the main process and was successfully performed^3^.

At this stage, we note that each NGH reservoir around the globe has its own distinctive geological characteristics, and that these need to be fully examined to determine compatible production techniques. These NGH geological characteristics also vary with formation depth and are determined by reservoir pressure and temperature. For example, the temperature and pressure ranges around 278 K and 70 bar on the Alaska North Slope. The NGH site in the Nankai Trough of Japan is located at a depth near 1000 m and ~287 K. In the UBGH site, the ranges of temperature and pressure are 288–293 K and 200–220 bar, respectively. The pressure exerted on an NGH site is determined by the hydraulic pressure exerted by the water column (depth), but the surrounding temperature may be much higher than expected because of a geothermal gradient of the NGH bearing sediment. The two exchange approaches using CH_4_-CO_2_ and CH_4_-CO_2_/N_2_ are to a certain degree adaptable, but in reality, we still need to develop a more feasible approach to deal with huge amounts of NGH at various conditions.

In this work, we demonstrate an NGH production method using air and CO_2_/air. Air is abundant and is available at any time and at any location, which makes it the most attractive element for practical production of NGH. It is known that N_2_ and O_2_ gases can play a role in breaking down methane hydrates because of the chemical potential difference (Δμ) between their gas and solid phases^16^,^17^,^18^. Earlier such methods for replacement and dissociation were only considered for use in systems with very dilute methane hydrates^13^,^14^,^15^,^16^,^17^,^18^. Accordingly, a continuous supply of fresh air into the NGH can make it melt completely. However, the methane concentration in the recovered gas becomes quite small, requiring additional separation facilities. The use of pure air contributes to methane production by NGH melting, but the key issue arises as to whether replacement can occur when we further extend the systems with concentrated methane hydrates or injection of CO_2_-enriched air (CO_2_/air). To address in detail the process of releasing methane from NGH using air or CO_2_/air, we defined the new variable ‘critical methane concentration' (CMC), which consists of the number of methane and injected molecules, and performed experiments to reveal the mechanisms involved (Fig. 1a).

## Results

The initial stage of air injection is intended to strongly decompose the NGH, release the methane and finally alter the gas-phase composition around the NGH. Meanwhile, the guest species of nitrogen and oxygen are expected to possess strong potential for forming pure, as well as mixed, hydrates. At this point, the following issue arises and needs to be addressed. How does air drive the NGH to melt? The fresh air might have the maximum potential for decomposing NGH, but gradually weakens and eventually loses its methane recovery capacity at a certain methane concentration. The NGH is continuously depleted until the chemical potentials of methane in both the solid NGH and gas phases are equal, and right after an arbitrary point, decomposition of methane hydrate stops and anomalous preservation of the hydrate phase occurs.

Now, we define the ratio between the methane concentration in hydrate (nCH_4_) and the injected gaseous air (or CO_2_/air; nair or nCO_2_/air) at equal chemical potentials as the ‘critical methane concentration' (CMC). The CMC plays a central role in determining methane recovery from NGH and can be influenced by complex surrounding factors such as the depth of marine NGH, geothermal gradient, sediment geochemistry and amount of saturated pore water.

**Figure 1:**
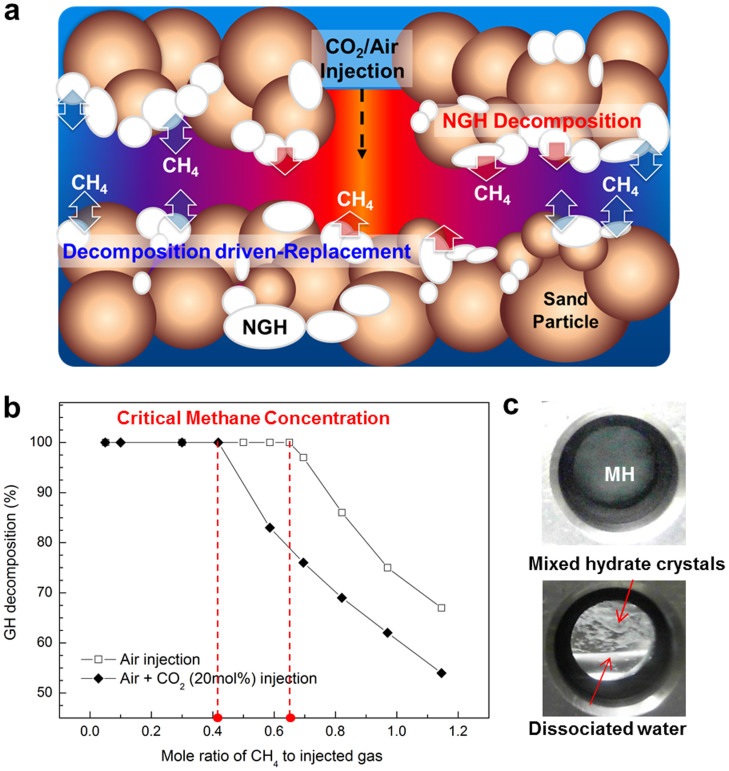
NGH production method using air and CO_2_/air. (a) Schematic illustration of the described air (or CO_2_/air) driven NGH production method composed of two distinct steps: NGH decomposition caused by injected air (or CO_2_/air); subsequent decomposition driven guest exchange process. (b) Discovery of the concept of critical methane concentration (CMC) depending on the molar ratio between CH_4_ and injected air and CO_2_/air. (c) Methane hydrate at 288.15 K and 200 bar before air injection (top), and mixed hydrate crystals coexist with water at 288.15 K after air injection above the critical concentration (bottom).

For precise CMC analysis we, above all, limited the experimental scope to the achievement of either complete or partial NGH decomposition. We used a fixed-volume high-pressure reactor, controlling the molar ratio between methane in the NGH and air (nCH_4_/nair) according to the initial NGH amount. The temperature was fixed at 288.15 K and air was injected at 200 bar to match closely the conditions found in the UBGH in the East Sea of Korea. When a large amount of air (nCH_4_ ≪ nair) was present, complete NGH decomposition occurred (Fig. 1b, left part of dotted line), thus leading to NGH depletion. In other words, the continuous supply of fresh air to NGH will make it possible for the gaseous phase to be in a more CH_4_-dilute state, maintaining its value lower than the CMC, and thereby favorably leading to full decomposition of methane hydrates. Here, it becomes quite interesting to see what occurred above the CMC. We observed the partial NGH decomposition (Fig. 1b, right part of dotted line). Thus, we identify three phenomenological stages: release of methane molecules from NGH, NGH structure destruction via melting and increase in the concentration of gaseous methane, which are all caused by the chemical potentials of guest-molecules. Approaching the dynamic equilibrium between solid NGH and gaseous air-methane, the vigorous escape of methane from NGH is suppressed and eventually ceases. Accordingly, it is interesting to determine the lowest methane concentration (CMC) that limits methane production from NGH. The complete NGH decomposition at the CMC can readily be confirmed visually, but above the CMC, a noticeable amount of NGH still remains (Fig. 1c).

**Figure 2:**
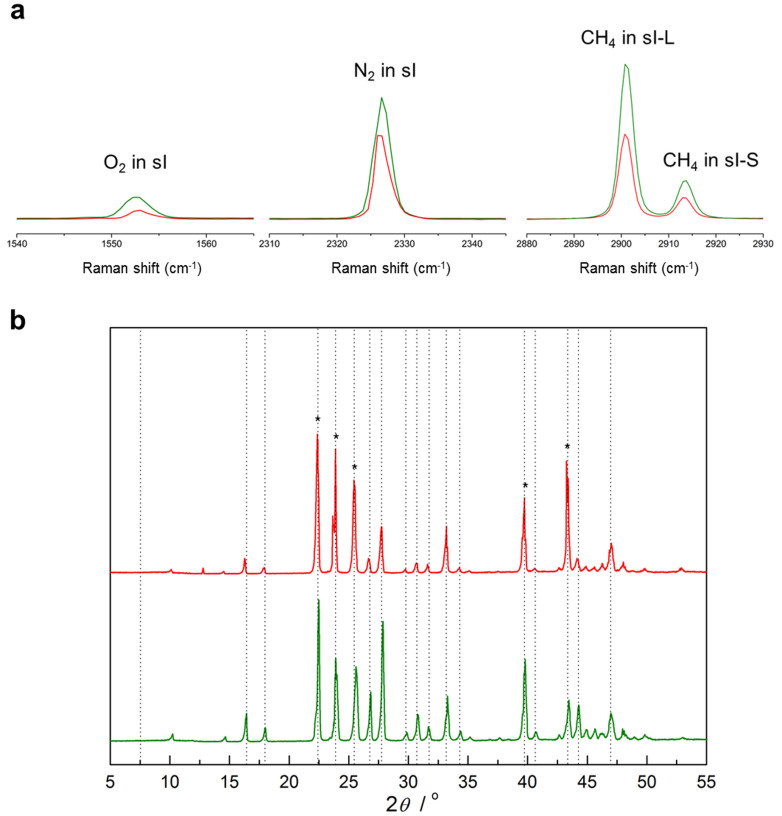
Formation of mixed sI hydrate beyond the critical molar ratio. Red line represents the hydrate phase when methane-air mole ratio is 2.01. Green line corresponds to the hydrate phase when the ratio is 5.65. (a) The Raman spectra of the mixed N_2_ + O_2_ + CH_4_ hydrate at 77 K. (b) PXRD patterns of each hydrate at 93 K. Asterisks in (b) indicate reflections from hexagonal ice (I_h_) phase.

When the ratio of CH_4_-air is greater than CMC, some methane hydrates dissociate and water is produced. Simultaneously, the formation of gas hydrate is initiated by the gas mixture of injected gas and released CH_4_. Finally, there is methane contained in the hydrate phase but it is inseparable between initially existent CH_4_ and reformed CH_4_ in hydrate. To obtain more detailed structural and compositional information above the CMC, NGH samples remaining after sufficient exposure to air (nCH_4_ ≫ nair) have to be carefully quenched (see Methods) and analyzed for clear identification of solid phases that might form with the aid of air. The Raman spectra (Fig. 2a) of two samples formed from different methane-air mole ratios (>CMC) reveal that the injected N_2_ and O_2_ molecules are continuously encaged in NGH until the equilibrium state is reached^19^,^20^. Stable capture of air components in sI-S and sI-L strongly implies that NGH decomposition, after gas injection, causes chemical replacement even at high temperatures, in the UBGH condition. In addition, the corresponding PXRD patterns (Fig. 2b) confirm that a mixed hydrate structure remains in the Cubic Structure I (sI) phase. These two spectroscopic results suggest that guest-replacement is likely to occur beyond the CMC. The water coming from partial dissociation of the hydrate by air injection is represented as hexagonal ice (I_h_) because the PXRD patterns were analyzed at 93 K. Quantitative analysis was conducted to find the relative contributions of the two key mechanisms, decomposition and replacement. The main peak intensities were determined by profile-matching the whole-pattern within the space group corresponding to structures ice I_h_ and sI.

**Figure 3:**
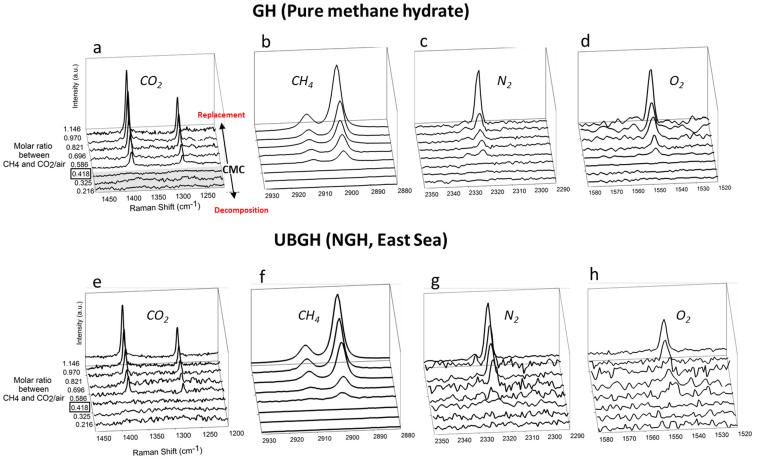
Direct visualization of guest-exchange analyzed by the Raman spectra after the injection of CO_2_/air (20 mol% CO_2_ and 80 mol% air) into pure CH_4_ hydrate (a–d) and NGH (e–h). CO_2_/air injection was performed at 288.15 K and 200 bar. As the CH_4_-CO_2_/air ratio increased, significant encapsulation of CO_2_, N_2_, and O_2_ molecules in the hydrate cages were detected. (a, e) C-O stretching and bending vibrational modes of CO_2_ molecules in the large cages of the hydrate; (b, f) C-H stretching vibrational modes of CH_4_ molecules in the hydrate; (c, g) N-N stretching vibrational modes of N_2_ molecules in the hydrate; (d, h) O-O stretching vibrational modes of O_2_ molecules in the hydrate.

**Figure 4:**
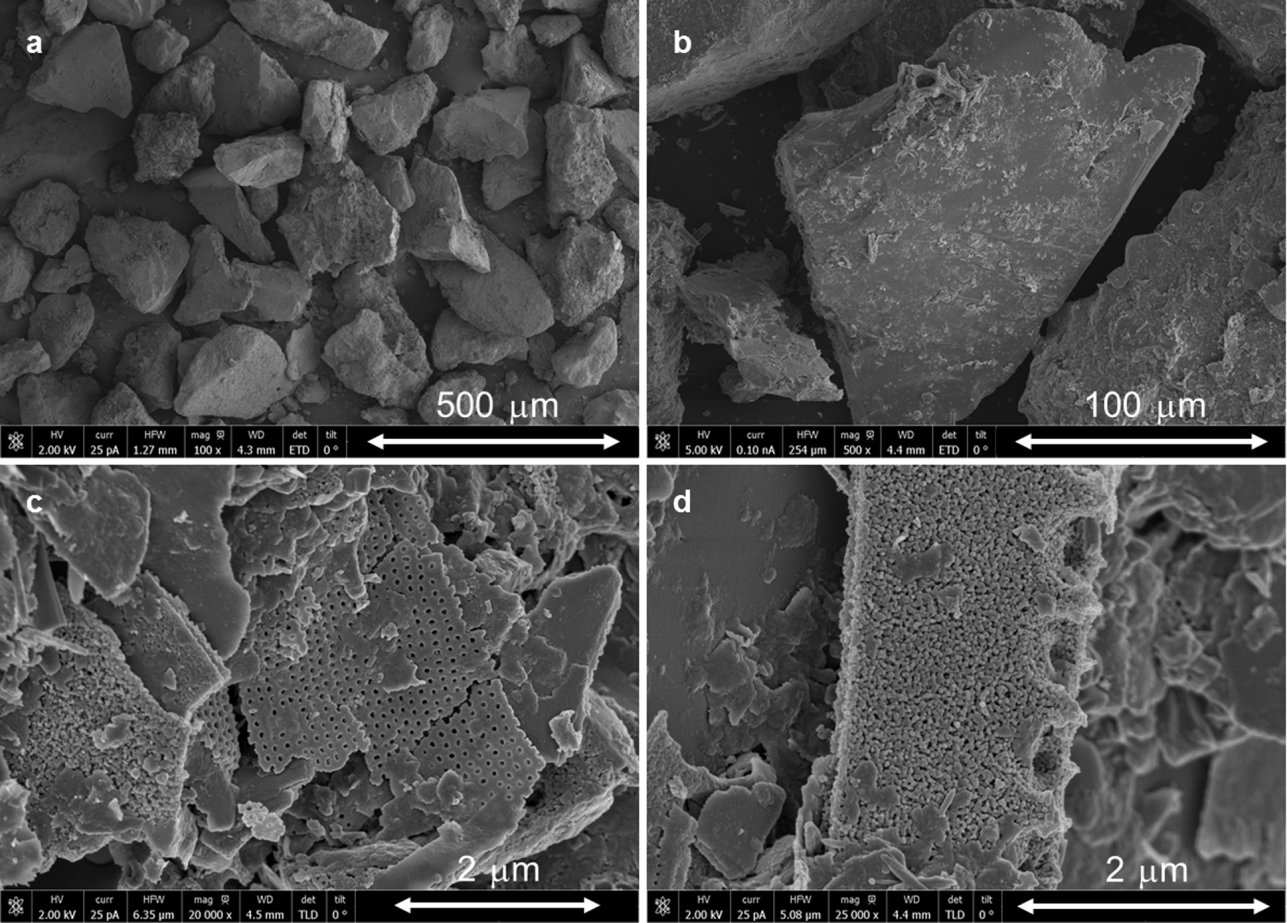
SEM images on dry sediments selected from the core section UBGH2-6-C.

Now we will focus on extending the phenomenological concept of air-based NGH decomposition to the CH_4_-CO_2_/air approach. Carbon dioxide is known to be a strong hydrate former and thus carbon dioxide mixed with air (20 mol% of CO_2_) slows the NGH decomposition rate. We note that the CMC is dramatically lowered to 0.418 when CO_2_ is added as a second guest (solid diamond in Fig. 1b). Fig. 3 shows the incorporations of N_2_, O_2_ and CO_2_ into the gas hydrate structure when the ratio of CH_4_-CO_2_/air exceeds CMC. Furthermore, we conducted the same experiment using real UBGH samples from the core section of UBGH-2-6-C. SEM images of the sediments are presented in Fig. 4. When the CH_4_-to-CO_2_/air ratios are greater than 0.418, which is the CMC for CO_2_/air (20 mol% of CO_2_), incorporation of gases in the gas hydrate structure is observed in the both pure gas hydrate system (Fig. 3a–d) and the UBGH system (Fig. 3e–h). Each of eight figures is presented in Supplementary Fig. S1–8.

**Figure 5:**
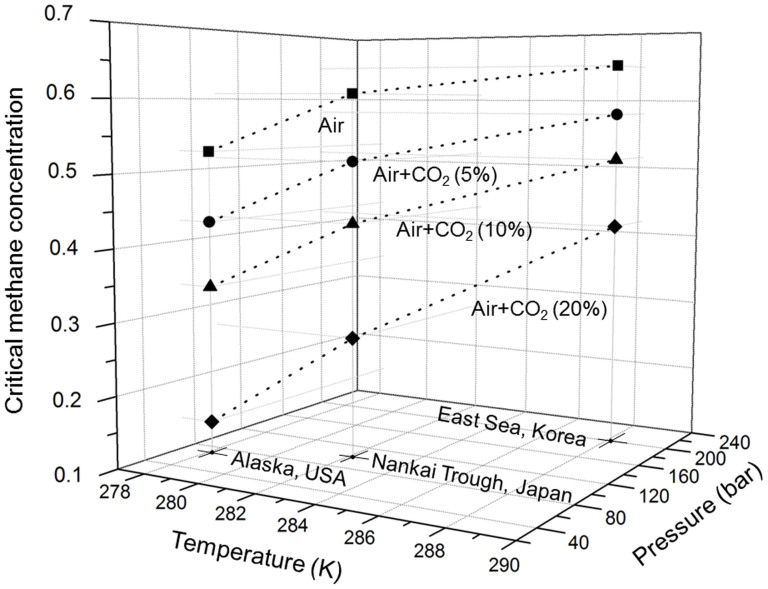
CMC observed in the three representative NGH reservoirs with different CO_2_ concentrations. Solid squares indicate pure air injection. Solid circles, triangles and diamonds indicate 5, 10 and 20 mol% CO_2_ in air gas, respectively. The dotted lines are the guides to the eyes.

Another important aspect of this research was to explore the effect of temperature and pressure on CMC-related behavior. We demonstrated the potential applicability of air and CO_2_/air injection to three NGH production test sites on the Alaska North Slope (USA), Nankai Trough (Japan) and Ulleung Basin (Korea), and the results are shown in Fig. 5. Representative pressures and temperatures from those sites were specified for the upcoming CMC analysis. To see the degree of NGH decomposition, we first injected pure air into the NGH and found a relatively high CMC of 0.53–0.65. The CMC gradually decreased as the NGH pressure and temperature decreased. It is again worth noting that the injection of pure air and CO_2_/air can simultaneously drive the two distinct mechanisms (decomposition and replacement) needed for NGH recovery. Through such a concept (combined hybrid production), we need to establish the process boundaries of the predominant mechanisms occurring in real NGH fields. When 5 mol% of carbon dioxide is added to pure air, its CMC shows a reduction of 10–18% compared with pure air alone, depending on surrounding conditions at the NGH sites. Several more tests revealed that the CMC generally decreases with increasing CO_2_. The lowering of the CMC by the activity of carbon dioxide results in a noticeable reduction of the NGH decomposition rate, and is probably linked closely to its potential for guest-exchange with methane. We thus conclude that use of high-CO_2_ in air leads to replacement-dominant NGH recovery.

Here, we notice that both CO_2_ and CH_4_ form sI hydrates, while air composed of N_2_ and O_2_ forms sII structures. Thus, we need to carefully analyze the structural patterns resulting from the complex interactions of the injected guest species of CO_2_, N_2_ and O_2_. According to the PXRD pattern (see Supplementary Fig. S9a), the sII hydrates prevail in 1 mol% CO_2_ in air gas, but in 3–20 mol% CO_2_, sI hydrate formation becomes possible. For verification of stability we measured three phase (H + L + V) equilibria using the isochoric process (see Supplementary Fig. S9b)^21^. Additionally, we observed that just a small increase in the carbon dioxide concentration in the vapor phase causes a significant increase of it in the mixed CO_2_/air hydrate phase (see Supplementary Table S1).

**Figure 6:**
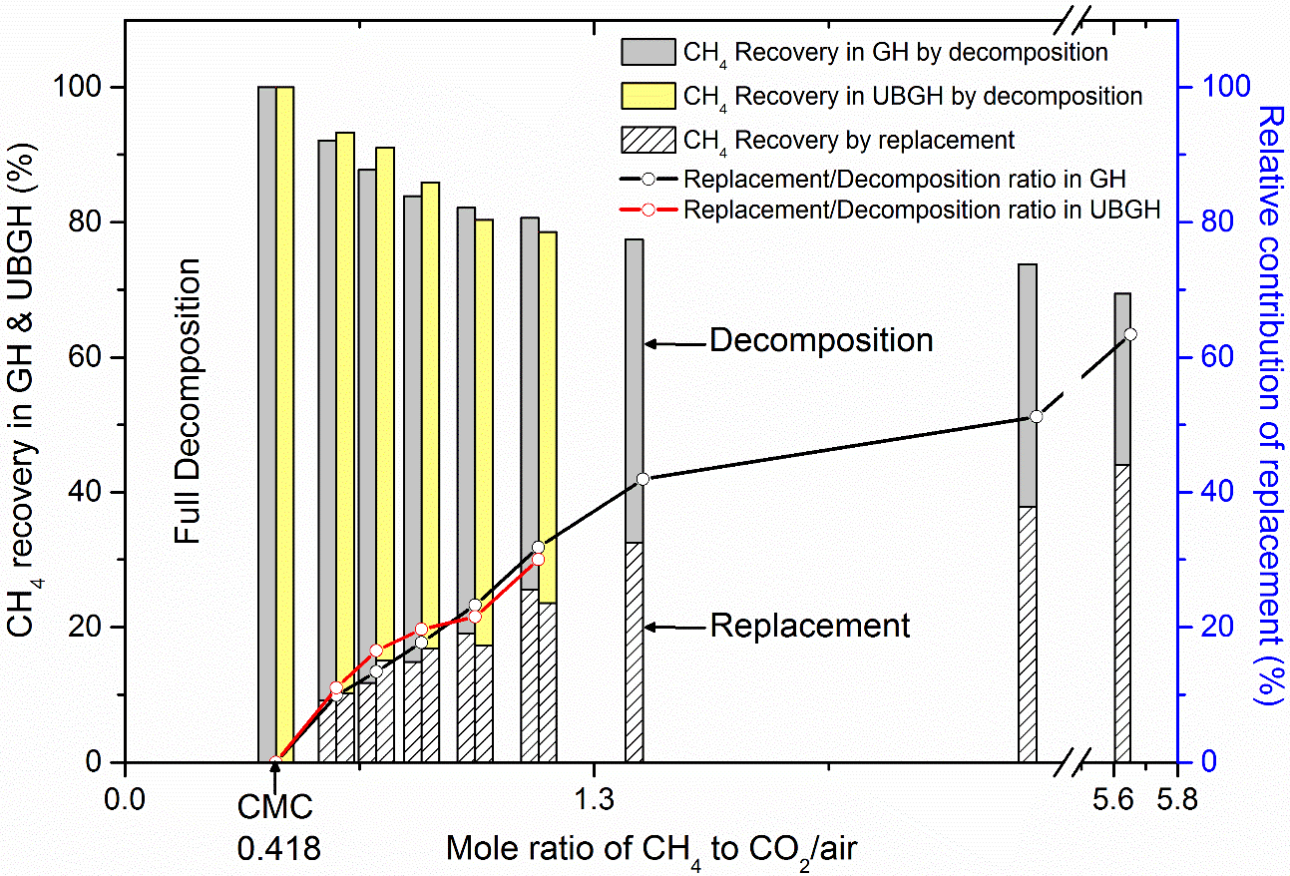
Decomposition/replacement ratio beyond the CMC observed under the conditions in the East Sea using CO_2_/air (20 mol% CO_2_ and 80 mol% air) gas. Black and yellow bars represent total CH_4_ recovery rates based on combination of decomposition and replacement in the pure GH and UBGH, respectively. When CH_4_ to CO_2_/air ratio exceeds the CMC, replacement between CH_4_ and CO_2_/air initiates. Shaded areas represent the fraction of replacement in the total CH_4_ recovery and solid lines represent relative contributions of replacement in each case.

To see patterns for mixed hydrates that have larger CH_4_-CO_2_/air mole ratios than at CMC, we carried out ex-situ HRPD experiments using the 9B beam line of the Pohang Accelerator Laboratory (see Supplementary Fig. S10). First, we determined the CMC of 0.418 in the ratio of CO_2_/air (2:8) and then observed the chemical exchange that occurred above the CMC. For comparison, the HRPD pattern at a CH_4_-air mole ratio of 2.53 is represented by the black line. From structural analysis we confirmed the coexistence of Structure I gas hydrate and hexagonal ice when either air or CO_2_/air was injected into the NGH and we obtained their relative amounts by entire-pattern fitting. The compositions of the sI gas hydrates were analyzed using GC. Thus the combined refined HRPD (ice to sI ratio) and GC results (replacement efficiency revealed by remaining gas composition in the hydrate phase) are shown as relative contributions of decomposition and replacement (Fig. 6). The detailed descriptions for the quantitative analysis can be found in Methods. CH_4_ recovery by decomposition is shown as gray bars and CH_4_ recovery by replacement is shown as shaded bars. The sum of these two bars is the rate of total CH_4_ recovery. Solid lines represent the ratio between ‘CH_4_ recovery by decomposition' and ‘CH_4_ recovery by replacement' in each sample. Interestingly, directly above the CMC, considerable amounts of CO_2_, N_2_, and O_2_ initiated replacement of CH_4_ in the NGH, even under harsh, deep-sea conditions. Particularly, we note that the contribution of replacement to NGH recovery (versus the contribution of decomposition) became more significant as the ratio of methane to CO_2_/air increased. Another notable feature is that the NGH production concept based on the CMC can be unrestrainedly applicable to a variety of geological settings with site-specific hydraulic pressures and geothermal gradients (red line in Fig. 6 shows UBGH sediment effect).

## Discussion

The formation of pure N_2_ or/and O_2_ hydrate requires harsh p-T conditions compared to the mild formation condition for pure CH_4_ hydrate. However, when the CH_4_ hydrates are pre-existing and gases are injected into the pre-existing CH_4_ hydrates, as the NGH reservoirs, the circumstance is different from that of pure gas hydrate formation condition. When pre-existing CH_4_ hydrates come into contact with the foreign gases such as air or CO_2_/air, slight decomposition of the CH_4_ hydrate is inevitable. Moreover, CH_4_ released from the pre-existing gas hydrates changes the composition of the gas phase (CH_4_ + N_2_ + O_2_ or CH_4_ + N_2_ + O_2_ + CO_2_ gas mixture), and eventually the change in gas composition causes the injected air or CO_2_/air gas to form the hydrates after all. This is why we introduced the concept of the ‘Critical Methane Concentration'. When the ratio of CH_4_ to air or CO_2_/air is less than or equal to the CMC, all CH_4_ hydrates dissociate. However, when the ratio is higher than CMC, partial decomposition of methane hydrate is observed and injected air or CO_2_/air form many gas hydrates. That is to say, we note that the phase equilibrium data of the injected gas is not the only criterion for determining whether or not the replacement can occur.

The most urgent task to advance NGH production is to find an economically feasible means that can proceed by spontaneous, natural processes. As a major future energy resource, NGH may compete with shale gas only if we can reduce the cost of primary production from large NGH deposits. Depressurization has been attempted in NGH field tests, most recently in the Nankai Trough (Japan). We understand that, by whatever method we choose, methane gas will be released from solid NGH by melting, by decomposition or by replacement via chemical exchange. A number of strongly influential variables that drive methane extraction control the two different NGH production mechanisms, while which of the two mechanisms is predominant remains to be determined.

From the insights gained from this study, we suggest a plausible strategy for extracting fuels from NGH. In the first phase, CO_2_ (transported from inland sources) and compressed air is mixed in-situ to prepare for injection into the NGH layer. This ‘in-situ mixing' concept allows a very effective preparation procedure for injecting gas mixtures. Liquid CO_2_ transported from the inland sources is then mixed with compressed air captured above the production test field to produce the CO_2_/air gas mixture. The CO_2_/air gas mixture is then injected into the sediment and diffused throughout the NGH layers. The first contact between the CO_2_/air gas mixture and the gas hydrates will induce decomposition of a certain portion of NGH in the sediments. This decomposition step eventually increases the partial pressure of CH_4_ inside the NGH layer and induces a ‘decomposition driven-replacement process'. The increased population of CH_4_ molecules significantly reduces the thermodynamic barrier for replacement reactions. This replacement process enhances the geo-mechanical stability of the NGH layer by forming stable hydrate lattices. Here, we again note that the nondestructive replacement process preserves host water-lattices in the NGH-bearing sediments as part of the newly formed air or CO_2_/air hydrates. In contrast, NGH decomposition by depressurization requires large volumes of water to be displaced from the NGH. Furthermore, the ensuing endothermic temperature drop caused by decomposition can result in undesirable formation of secondary methane hydrate or ice in the well and near the wellbore. The chemical exchange of dissimilar guest molecules does not involve significant cooling^22^, and thus the geo-mechanical stability of gas hydrate-bearing sediments is less threatened.

We present three key aspects of our findings: 1) We defined a new factor (CMC) related to the methane recovery rate from natural gas hydrates, 2) We obtained CMC values for three distinct conditions (simulating three representative reservoirs) experimentally, and 3) We verified that starting decomposition leads to replacement of methane by injected air or CO_2_/air in NGH. According to the present outcomes, we conclude that the proposed process is likely the efficient, nondestructive and commercially-viable means (compared to conventional ones) for onshore and offshore methane production from NGH, and it can be widely adapted to diverse geological conditions. We acknowledge that the CH_4_ cage occupancies of naturally occurring gas hydrates may differ depending on the surrounding conditions, such as the sediment type, the methane saturation percentage in the pore water, and the macroscopic morphology of the gas hydrate, among others. Therefore, to apply CMC fully in the field of gas hydrate production, general trends pertaining to the cage occupancy of target production sites are required.

## Methods

### Materials

Water of ultrahigh purity was obtained from a Millipore purification unit. CH_4_, air (O_2_ 21 mol% and N_2_ balance) and CO_2_/air gas were purchased from the Special Gas Company (Daejeon, Republic of Korea), with a stated minimum purity of 99.95 mol%.

### Sample preparation and gas injection procedures

To assess the effect of the molar ratio of CH_4_ and air (or CO_2_/air), we used a fixed-volume high-pressure stirred reactor. First, we charged the reactor with the desired volume of water and charged 200 bar of methane gas at 288.15 K. The formation of methane hydrate was achieved by decreasing the temperature in an isochoric condition. The heating-cooling cycle was repeated to confirm the complete conversion of water to methane hydrate. When the methane hydrate was fully formed, additional methane gas was supplied to recover pressure reduction due to hydrate formation. When the pressure and temperature were stabilized at the desired conditions (e.g., 200 bar and 288.15 K), methane gas in the reactor was rapidly exchanged with air (or CO_2_/air) using a syringe pump (ISCO, model 260D). To prevent the dissociation of methane hydrates during the gas exchange, we used a back-pressure regulator to maintain a constant pressure (e.g., 200 bar). Also, in order to eliminate the heating effect of air, air pre-cooled to a temperature of 288.15 K was used. When we performed the gas exchange of methane with air or CO_2_/air, we used another high-pressure reservoir cell in the same temperature-controlled bath (see Supplementary Fig. S11). The inner volume of the gas reservoir cell is 500 ml, and it served as a container for pre-cooled gas. During every exchange process, the container and an ISCO pump were pressurized 25% higher (20–50 bar) than each target pressure before the injection process. For example, we pressurized the precooled reactor and ISCO pump at 250 bar when the target pressure was 200 bar. The back pressure regulator was set at an exact target pressure. Before performing the gas exchange experiments, we pre-tested the gas exchange capacity of our experimental setup with the empty cell (50 ml) and confirmed the complete gas exchange (>99%) via GC. It takes 30 seconds completely to replace the CH_4_ in the reactor with air (or CO_2_/air). Once the volume of methane hydrate was obtained using the density of methane hydrate (930 kg m^−3^)^23^, the volume occupied by the injected gas became known, and the mole of the injected gas was calculated by applying an ideal gas law. In this way, the mole ratio between CH_4_ and air (or CO_2_/air) was determined. In case of the UBGH sample, the physical properties of porosity and grain density for sand were acquired from KIGAM (the UBGH drilling expedition team), which was 70% and 2650 kg m^−3^. The pressure and temperature during the entire process was monitored using a custom-built Labview program, and we periodically checked if the pressure and temperature had reached constant values. We initially assessed the decomposition of hydrate using a look-through quartz window on the high-pressure reactor. When the ratio of CH_4_ to air (or CO_2_/air) was smaller than the CMC, we confirmed the disappearance of the last hydrate crystal, as visual observation is widely used in determining the hydrate equilibrium conditions.

### Mixed-phase gas hydrate sampling using liquid nitrogen quenched helium gas

When the reaction of the gas hydrate and air (or CO_2_/air) was completed (confirmed by PT monitoring; finished within 72 hours), we took representative samples of the product for further spectroscopic analyses. To keep the original phase intact, we used rapid cooling method using liquid-nitrogen quenched helium gas and liquid nitrogen. If the gas in the reactor is vented to atmospheric pressure under ambient conditions, the original composition of the product would significantly change. Therefore, we rapidly exchanged the gas phase in the reactor with pre-cooled helium gases. Because helium retains its gaseous phase at the liquid-nitrogen temperature (~77 K), the gas phase in the reactor was effectively exchanged to helium gas at the near-liquid nitrogen temperature. At the same time, the reactor was immersed in liquid nitrogen to freeze the whole reaction product rapidly. In this way, we kept the original phase composition of the product during the sampling procedure. When the product was completely frozen, it was ground into particles of less than 200 microns, and they were analyzed using spectroscopic tools (XRD/HRPD, Raman) and gas chromatography.

A quantitative analysis for decomposition/replacement beyond the critical methane concentration was performed by a combination of XRD/HRPD and GC results. When mole ratio of CH_4_ to CO_2_/air was greater than the CMC, the solid phase (mixed gas hydrate) and water coexisted. These products were acquired by a rapid sampling method, and water phase turns into ice phase. Therefore, the amount of the ice phase corresponds to the amount of decomposition. The recovery by replacement was determined by a GC analysis of the hydrates using same method used in the literature^13^,^14^,^15^. In this way, the total CH_4_ recovery and relative CH_4_ recovery depending on the ratio of CH_4_ to CO_2_/air were calculated.

### Spectroscopic analysis

For Raman measurements we used the Horiba Jobin Yvon LabRAM HR UV/Vis/NIR high resolution dispersive Raman microscope in which a CCD detector was equipped and cooled by liquid nitrogen. Samples were kept at 77 K during measurements. The excitation source was an Ar-ion laser emitting a 514.53 nm line. The laser intensity was typically 30 mW.

The XRD patterns were obtained using a Rigaku D/max-IIIC diffractometer with CuKα as a light source (λ = 1.5406 Å) at a generator voltage of 40 kV and a generator current of 300 mA. A low-temperature stage attached to the XRD unit maintained the working temperature at 93 K, and a step-scan mode was applied (0.02° step-size and 3 s per step).

The HRPD patterns were collected using PAL (Pohang Accelerator Laboratory, Republic of Korea) Synchrotron. During the measurements, the θ/2θ scan mode with a fixed time of 3 s and a step size of 0.005° for 2θ = 0 ~ 120° and the beam line with a wavelength of 1.5472 Å were used for each sample. The loading of the samples was performed at 77 K to minimize possible sample damage.

The concentration of gaseous guest-molecules in the hydrate sample was measured using a Young-Lin 6000 (Young-Lin, Republic of Korea) gas chromatography. The samples were moved to a precooled reactor (~77 K) each time, and it was evacuated using a vacuum pump. The gas phase was analyzed after being held at room temperature until the hydrate samples completely dissociated. Helium was used as the carrier gas, and the GC column was maintained at 55 kPa and 353 K.

## Author Contributions

K.H., K.D.-Y. and L.H. designed the research; K.H. and K.D.-Y. performed the research; K.D.-Y. and K.H. analyzed the data and K.H., K.D.-Y. and L.H. wrote the paper.

## Supplementary Material

Supplementary Information Supplementary information
