# Supplementary material for: Nondestructive natural gas hydrate recovery driven by air and carbon dioxide
Source: Sci Rep. 2014 Oct 14;4:6616. doi: 10.1038/srep06616 (PMC4196106; doi:10.1038/srep06616)
Supplement: Supplementary Information [file srep06616-s1.pdf]

Supplementary Information for

Nondestructive natural gas hydrate recovery driven by air and carbon dioxide

Hyery Kang[1,a], Dong-Yeun Koh[1,a], and Huen Lee[1,2*]

[1]Department of Chemical and Biomolecular Engineering, Korea Advanced Institute of Science and Technology, 291 Daehak-ro, Guseong-dong, Yuseong-gu, Daejeon 305-701, South Korea

[2]Graduate School of EEWS, Korea Advanced Institute of Science and Technology, 291 Daehak-ro, Guseong-dong, Yuseong-gu, Daejeon 305-701, South Korea

[a]Both authors contributed equally to this work.

Correspondence and requests for materials should be addressed to H. L. (hlee@kaist.ac.kr)

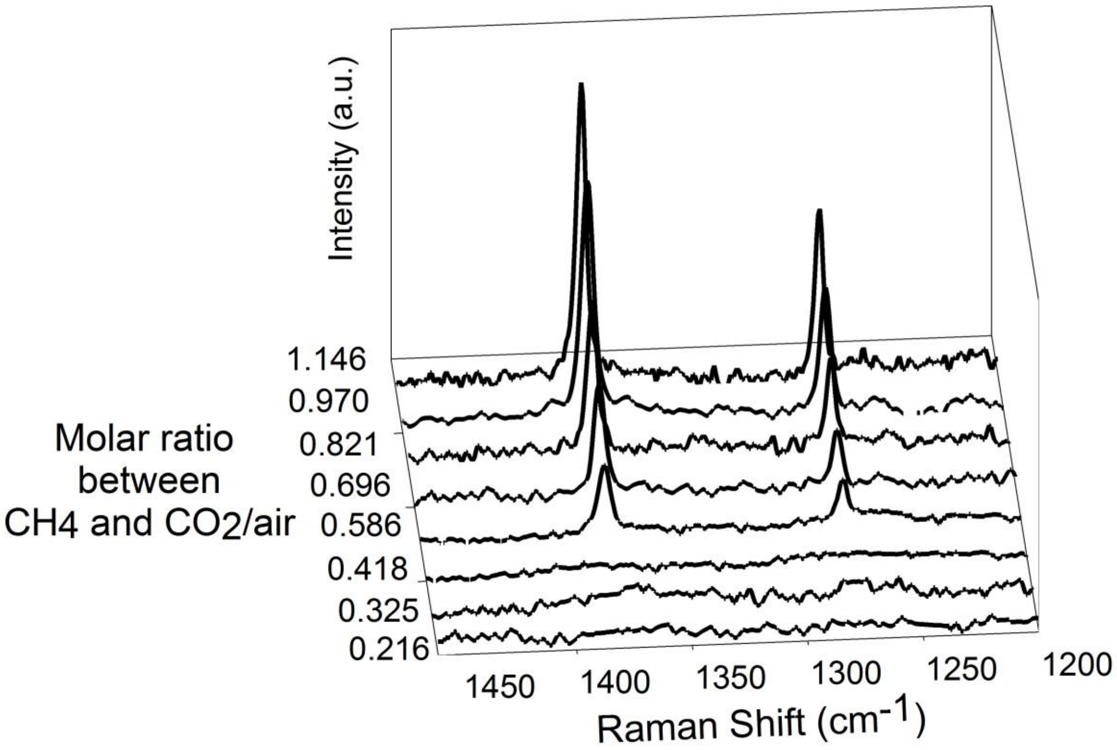

Supplementary Figure S1 | CO_2 in GH. C-O stretching and bending vibrational modes of CO_2 molecules in the large cages of the hydrate.

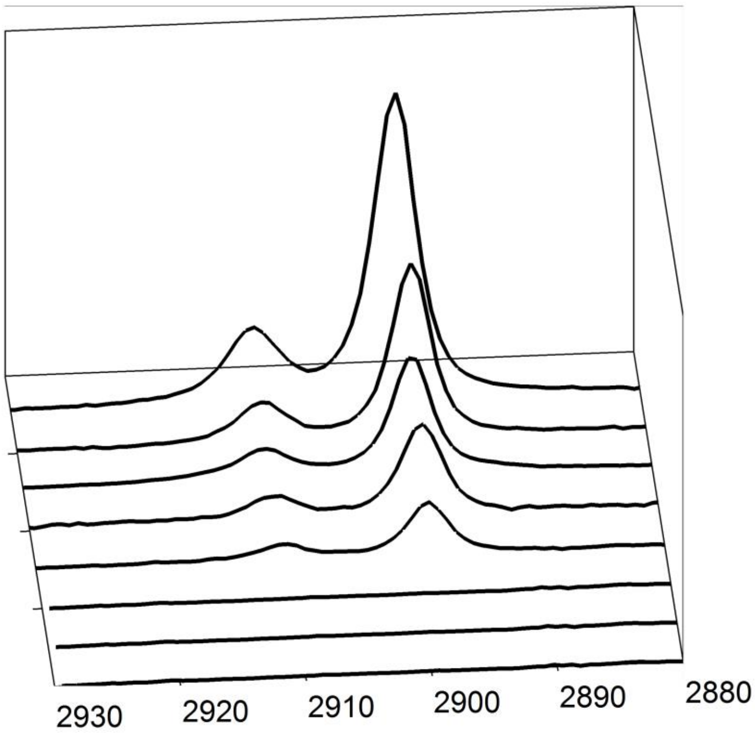

Supplementary Figure S2 | CH_4 in GH. C-H stretching vibrational modes of CH_4 molecules in the hydrate.

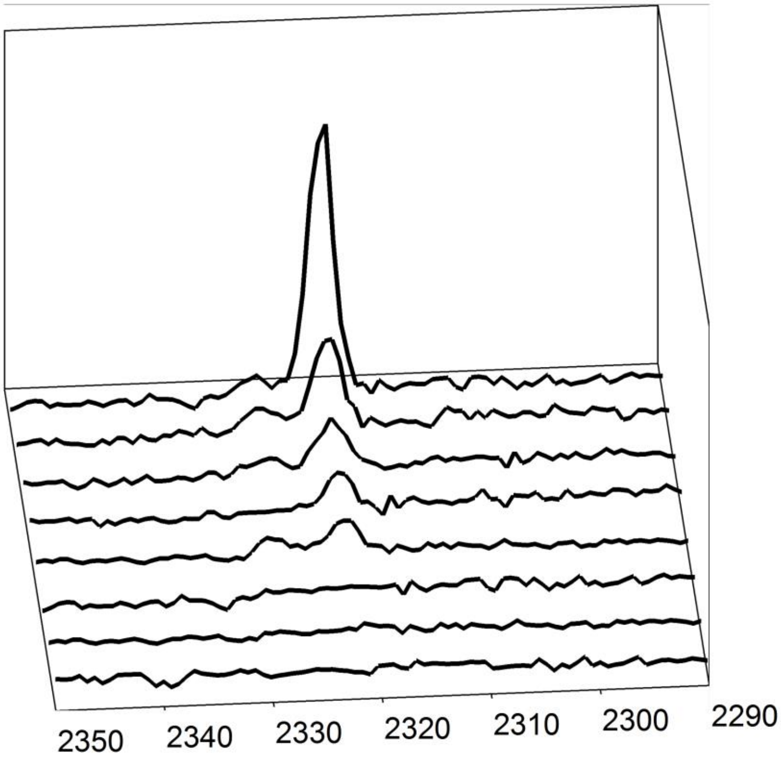

Supplementary Figure S3 | N_2 in GH. N-N stretching vibrational modes of N_2 molecules in the hydrate.

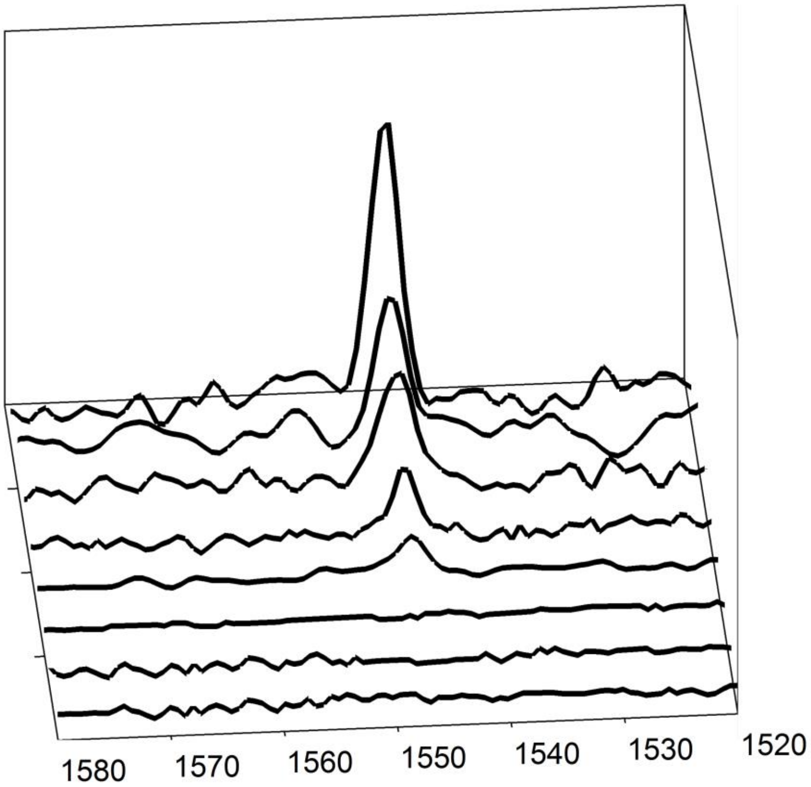

Supplementary Figure S4 | O_2 in GH. O-O stretching vibrational modes of O_2 molecules in the hydrate.

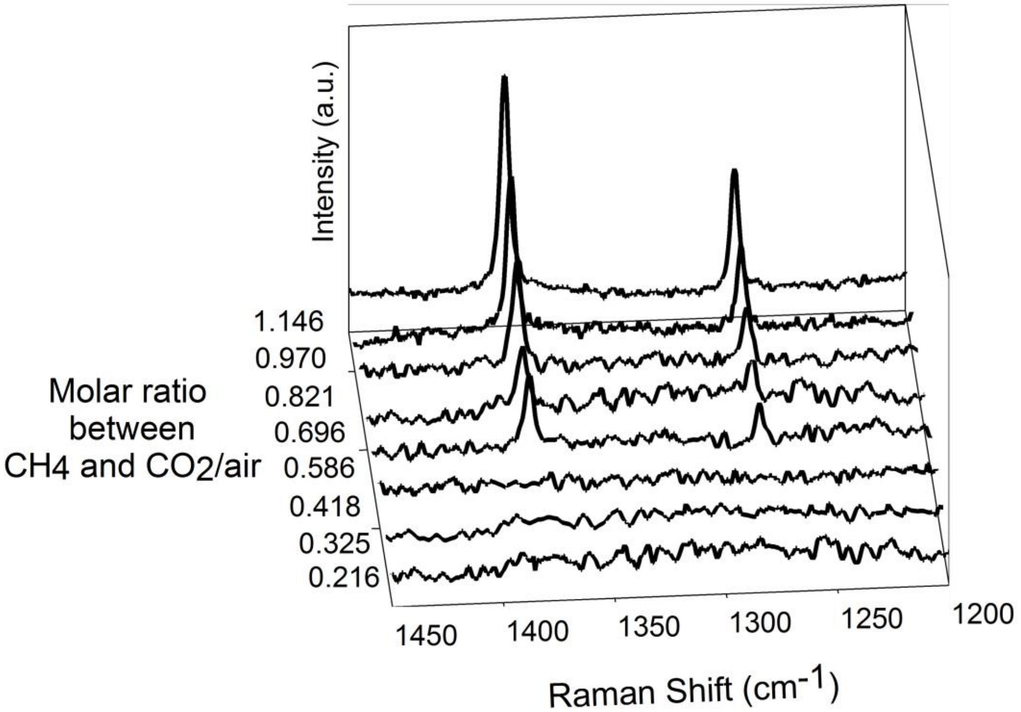

Supplementary Figure S5 | CO_2 in UBGH. C-O stretching and bending vibrational modes of CO_2 molecules in the large cages of the hydrate.

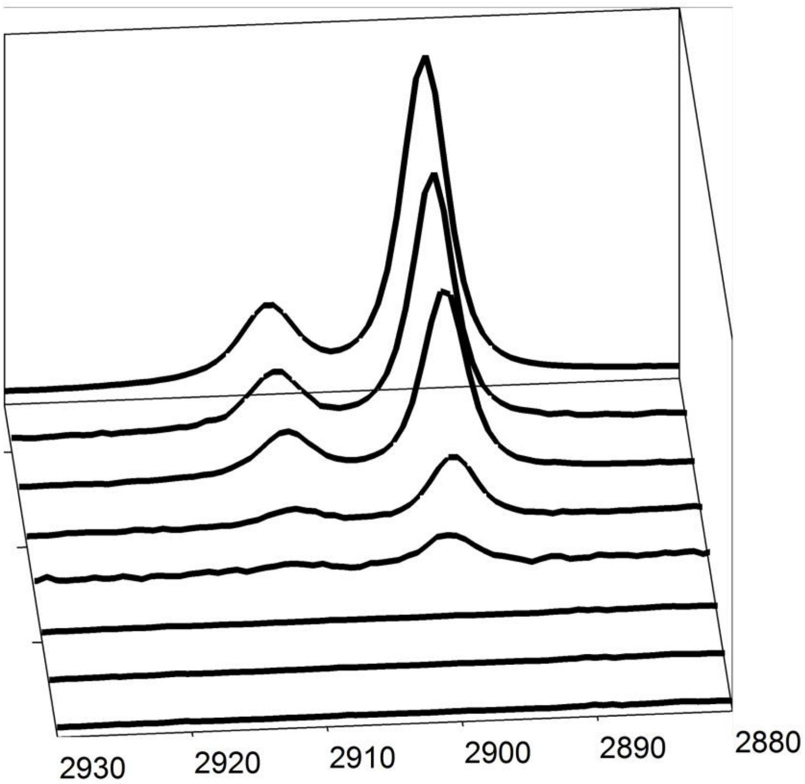

Supplementary Figure S6 | CH_4 in UBGH. C-H stretching vibrational modes of CH_4 molecules in the hydrate.

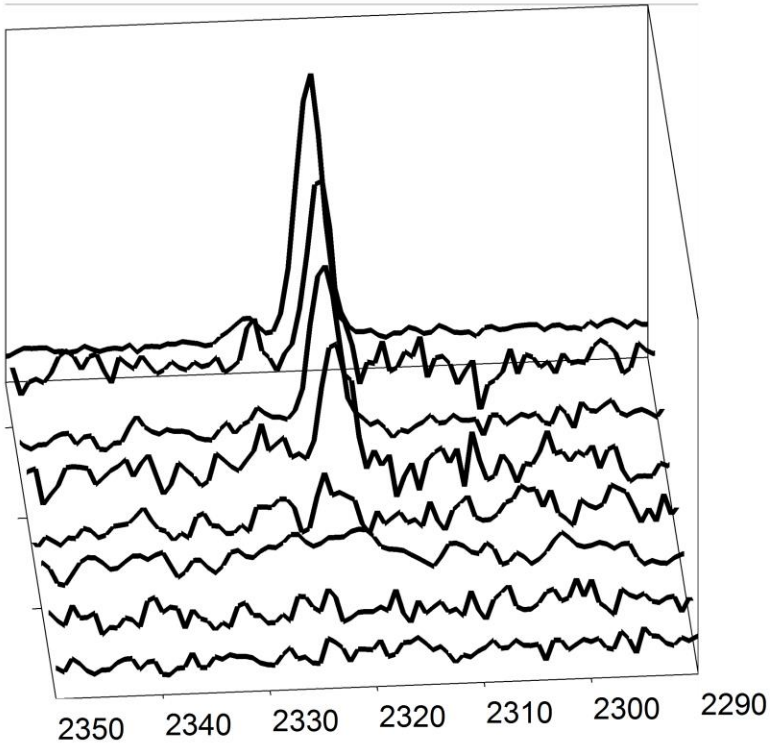

Supplementary Figure S7 | N_2 in UBGH. N-N stretching vibrational modes of N_2 molecules in the hydrate.

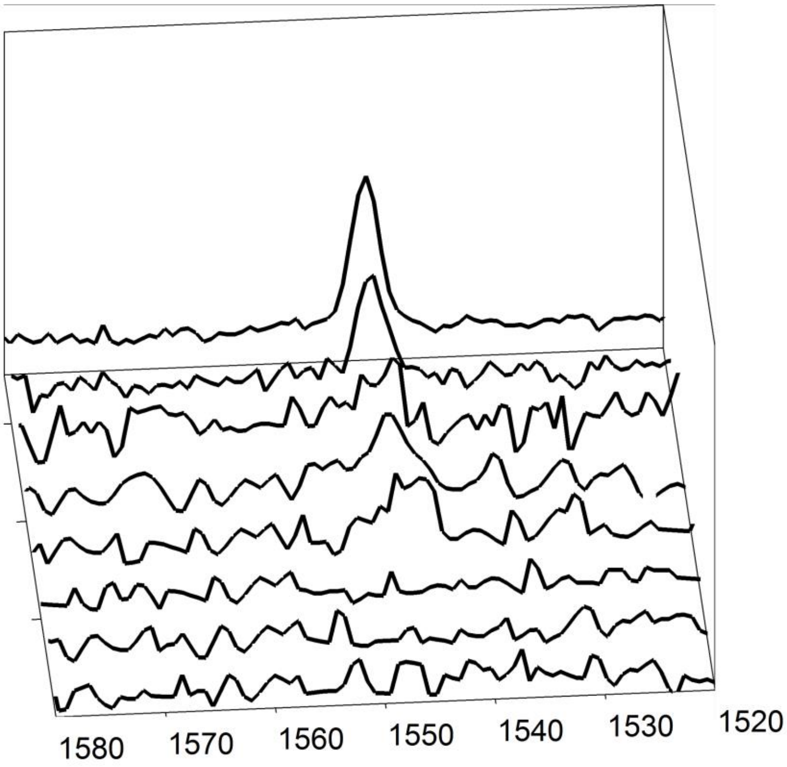

Supplementary Figure S8 | O_2 in UBGH. O-O stretching vibrational modes of O_2 molecules in the hydrate.

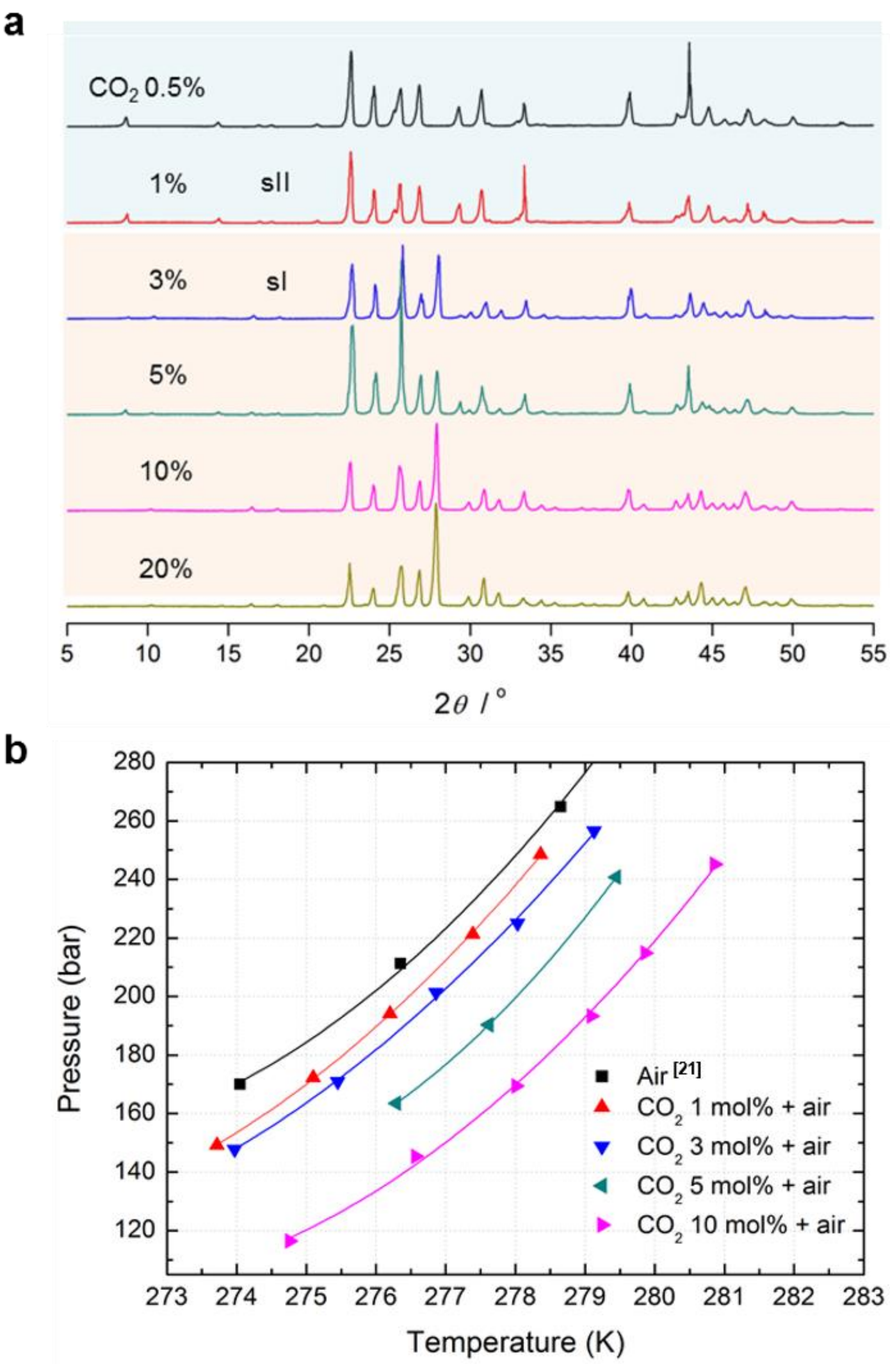

Supplementary Figure S9 | Hydrate formation with three gaseous guests of CO_2, N_2, and O_2. (a) Powder X-ray diffraction patterns of three gaseous guests (CO_2, N_2, and O_2) of hydrates having different CO_2 concentrations. (b) H-Lw-V phase equilibrium data for the CO_2 + air (O_2 20 mol% and N_2 balance) + water mixture measured at 1, 3, 5, and 10 mol% of CO_2.

Supplementary Table S1 | Gas composition in hydrate depending on CO_2 concentration in gas phase.

CO_2 concentration in CO_2/air gas (CO_2 %)	Gas composition in hydrate phase (%)	
	Air	CO_2
1	96.26	3.74
3	85.53	14.47
5	77.58	22.42
10	59.88	40.12
20	42.48	57.52

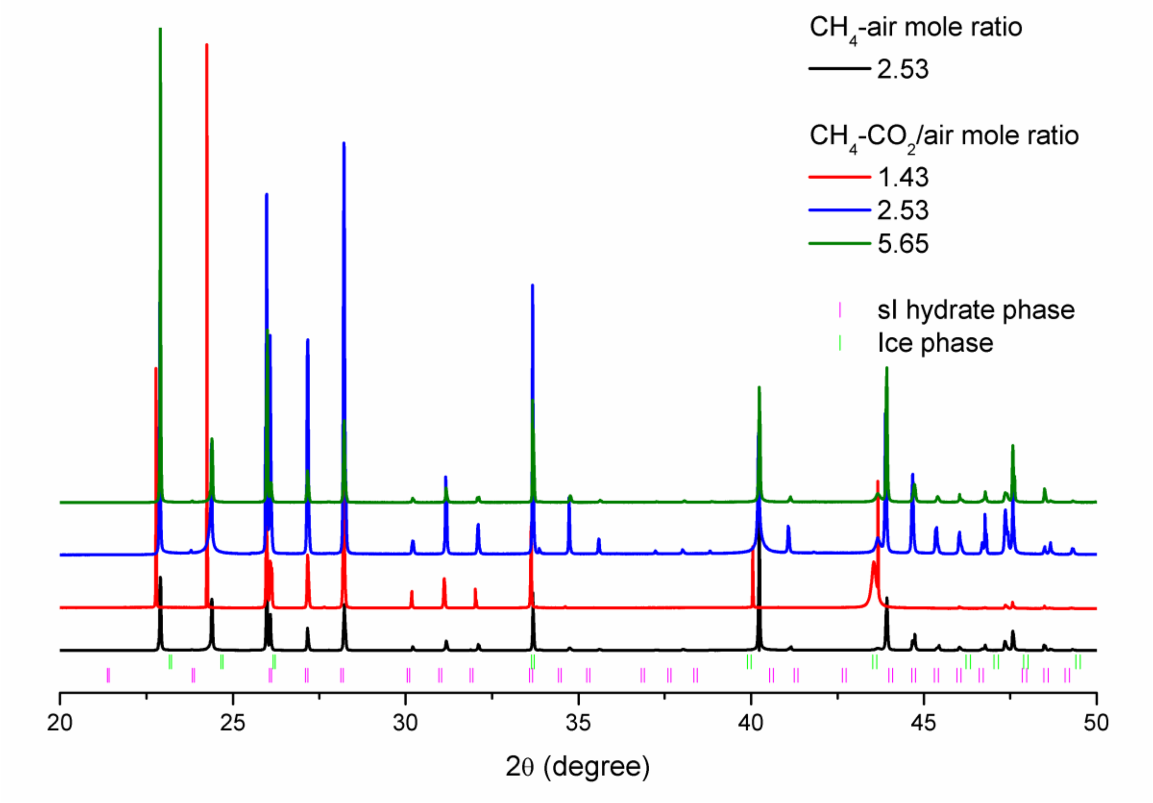

Supplementary Figure S10 | Synchrotron HRPD patterns at an equilibrium state having various methane-CO_2/air (2:8) mole ratios above the initial CMC of 0.418. Red, blue and green lines represent the HRPD patterns of 1.43, 2.53 and 5.65, respectively. For comparison, the HRPD pattern of the methane-air mole ratio of 2.53 is represented by black line (initial CMC was 0.65 for air injection).

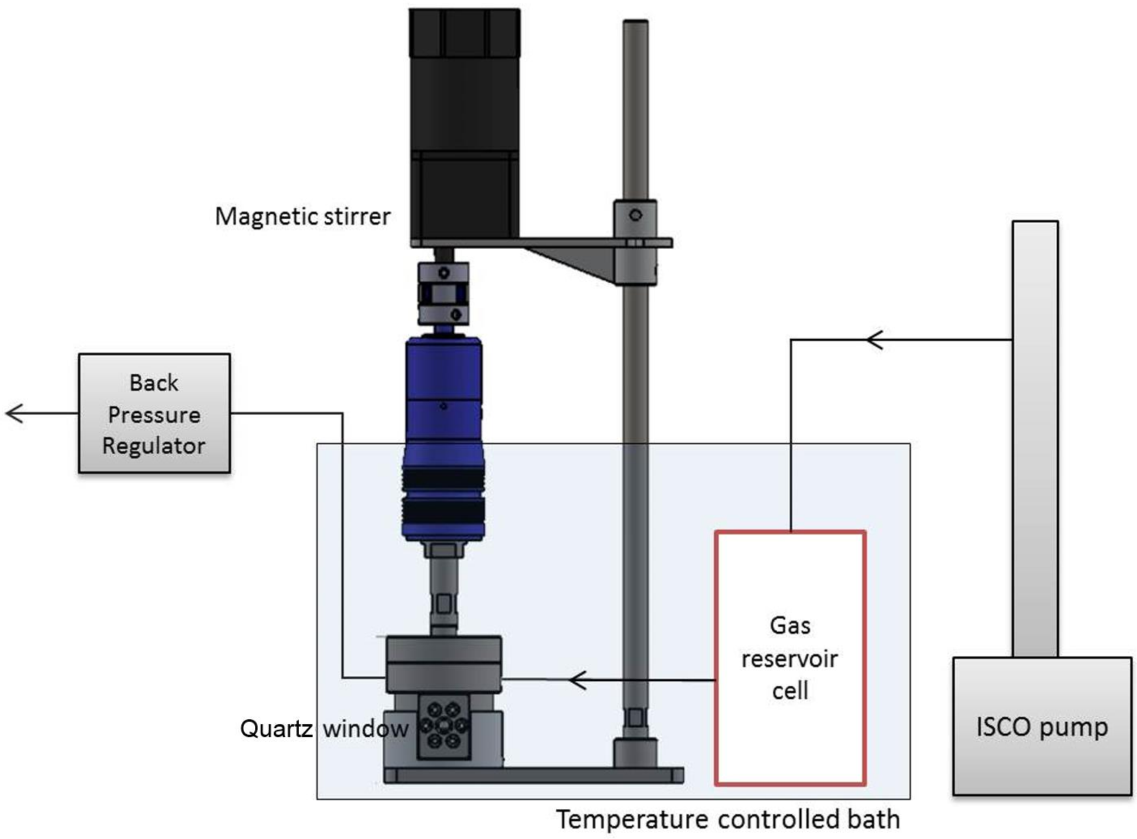

Supplementary Figure S11 | Schematic diagram of the experimental system.
